# Interface Chemistry and Reaction Pathway Regulation for Boosted Redox Kinetics in Aqueous Zn–S Batteries

**DOI:** 10.1002/advs.202513155

**Published:** 2025-11-05

**Authors:** Sibo Wang, Chen Li, Wanlong Wu, Guoli Zhang, Razium Ali Soomro, Wenchao Fu, Xiaoqi Sun, Bin Xu

**Affiliations:** ^1^ Institute of Advanced Energy Storage Materials and Technologies School of Chemistry and Chemical Engineering, Yan'an University Yan'an 716000 China; ^2^ Department of Chemistry Northeastern University Shenyang 110819 China; ^3^ National Frontiers Science Center for Industrial Intelligence and Systems Optimization Northeastern University Shenyang 110819 China; ^4^ State Key Laboratory of Organic–Inorganic Composites Beijing Key Laboratory of Electrochemical Process and Technology for Materials, Beijing University of Chemical Technology Beijing 100029 China

**Keywords:** aqueous zinc‐sulfur batteries, reaction pathway, redox kinetics, solid‐electrolyte interphase, tetramethylurea

## Abstract

Aqueous Zn–S batteries are promising candidates for large‐scale energy storage applications due to their high specific capacity and energy density. However, their performance is extremely plagued by the sluggish redox kinetics. Here, an interface chemistry regulator is proposed for both electrodes to facilitate reaction kinetics and promote stability. The tetramethylurea (TTMU) is selected as the electrolyte additive. It first preferentially adsorbs on the sulfur cathode surface and coordinates to Zn^2+^, thereby altering their reaction pathway. This reduces the energy barrier and promotes uniform ZnS nucleation, which accelerates reaction kinetics. At the same time, the additive induces an effective solid‐electrolyte interphase on the anode and enhances the reversibility and stability of Zn plating/stripping. With the help of 10% TTMU additive, the Zn–S battery delivers a high capacity of 1620 mAh g^−1^ with a low overpotential of 0.37 V at 0.1 A g^−1^, which is superior to 1138 mAh g^−1^/0.65 V in the benchmark Zn(OAc)_2_/ZnI_2_ electrolyte. With the increase of current density to 5 A g^−1^, the additive also significantly enhances the capacity from 48 to 913 mAh g^−1^. Promoted cycling stabilities are further achieved for both Zn electrode and Zn–S cells in the TTMU containing electrolyte.

## Graphical abstract

The reaction properties of both electrodes in aqueous Zn‐S batteries is regulated by a tetramethylurea electrolyte additive. It preferentially adsorbs on the sulfur surface and coordinates to Zn^2+^, thereby altering their reaction process. The energy barrier is reduced and nucleation is promoted, which accelerates reaction kinetics. It also induces an SEI layer on the anode, enhancing Zn plating/stripping reversibility. This strategy significantly boosts the electrochemical performance of Zn‐S batteries.

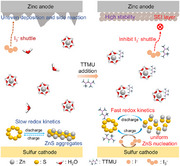

## 1 Introduction

Rechargeable aqueous zinc batteries are promising candidates for grid‐level energy storage,^[^ ^1^, ^2^, ^3^, ^4^ ^]^ as Zn metal anode offers high theoretical capacity (5845 mAh cm^−3^, 820 mAh g^−1^), low redox potential (−0.76 V vs SHE), and low cost.^[^ ^5^, ^6^, ^7^, ^8^ ^]^ Significant progress has been made in intercalation‐type cathode materials such as manganese oxides, vanadium oxides, polyanion compounds, and organic materials.^[^ ^9^, ^10^, ^11^, ^12^, ^13^, ^14^ ^]^ However, their theoretical capacities are typically below 600 mAh g^−1^, limited by the electrochemically inactive skeletons in their lattices. In comparison, elemental cathodes with conversion mechanisms provide higher capacities, offering promising paths toward high‐energy‐density devices. Sulfur is considered as a desired candidate due to its cost‐effectiveness and multi‐electron transfer reaction with a high theoretical capacity of 1675 mAh g^−1^.^[^ ^15^, ^16^, ^17^, ^18^ ^]^ The study of aqueous Zn–S batteries initiated in 2020,^[^ ^19^, ^20^ ^]^ yet great challenges remain. Unlike in extensively studied non‐aqueous Li‐S systems, the sulfur cathode undergoes direct solid‐solid conversion in aqueous zinc cells, which suffers extremely sluggish redox kinetics that lead to significant voltage polarization, rapid capacity decay, and consequently hinder the electrochemical performance and practical applications of aqueous Zn–S batteries.

Several strategies have been proposed to enhance the electrochemical performance of sulfur cathodes in aqueous zinc batteries. Li et al. proposed I_2_ as a redox mediator to facilitate the conversion of ZnS to S during the charge process, thereby reducing the overpotential from 0.90 to 0.72 V.^[^ ^19^ ^]^ Subsequently, “cocktail optimized” electrolytes combining I_2_ with tetraglyme or ethylene glycol were further developed to enhance sulfur conversion and suppress side reactions in Zn–S cells.^[^ ^21^, ^22^ ^]^ However, these iodine‐based additives primarily facilitate the charge process, whereas the discharge overpotential remains relatively high. To address this, our group proposed a dual mediator of trimethylphenylammonium iodide. This approach converts the discharge pathway into a solid‐liquid‐solid process, simultaneously enhancing reaction kinetics and suppressing iodine shuttling to achieve higher coulombic efficiency.^[^ ^23^ ^]^ Alternatively, sulfur active material has been composited with Fe(CN)_6_^3−/4−^ redox couples or Fe‐N_4_ catalysts to facilitate the conversion reactions,^[^ ^24^, ^25^ ^]^ while using selenium‐sulfur solid solutions as a cathode has been demonstrated with improved conductivity and reactivity.^[^ ^26^ ^]^ Despite these advances, the electrochemical performance of aqueous Zn–S batteries is still far from satisfactory, which calls for innovative strategies to overcome existing limitations.

Herein, we present an interface chemistry regulation strategy for both electrodes in Zn–S cells. The tetramethylurea (TTMU) with a favorable coordination site is selected as the electrolyte additive. Experimental and theoretical studies demonstrate that it preferentially adsorbs on the sulfur cathode surface, coordinates with Zn^2+^, and alters their reaction process. The reaction barrier is therefore reduced and nucleation is promoted, which enhances the reaction kinetics of the sulfur cathode. Meanwhile, the TTMU additive also induces an organic–inorganic hybrid solid‐electrolyte interphase (SEI) on Zn anode. It enhances the reversibility and stability of Zn plating/stripping. Upon the addition of 10% TTMU to Zn–S cells, the capacity of the cathode increases significantly from 1138 to 1620 mAh g^−1^, together with the reduced overpotential from 0.65 to 0.37 V. The cycle life is also promoted for both Zn electrode and Zn–S cells.

## 2 Results and Discussion

**Figure 1:**
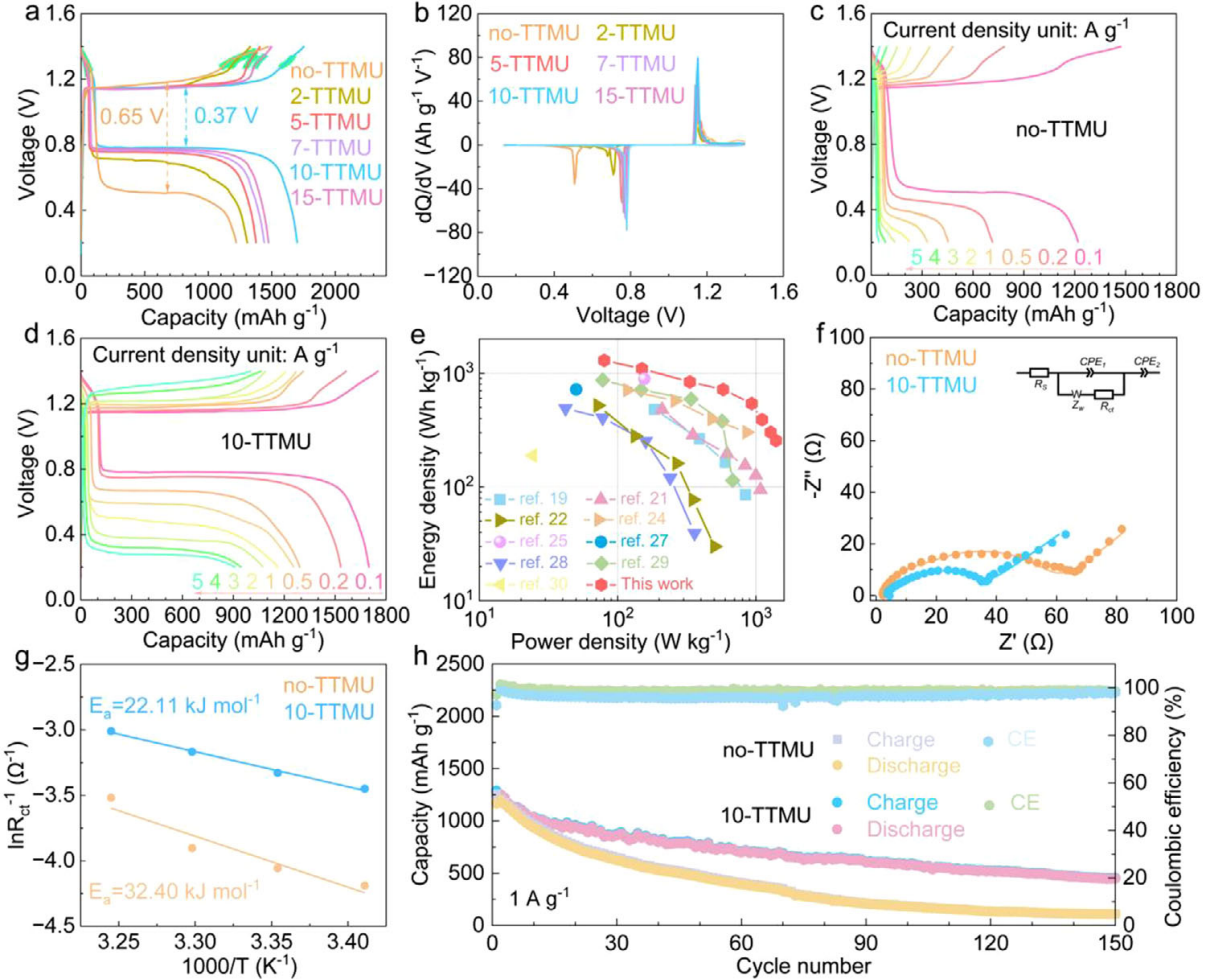
Electrochemical performance of Zn–S cells with the benchmark or TTMU containing electrolytes: a) charge/discharge curves (with iodine reactions shaded in green) and b) differential capacity curves at a current density of 0.1 A g^−1^; charge/discharge curves at different current densities in the c) no‐TTMU and d) 10‐TTMU electrolytes, and e) the Ragone plots with the 10‐TTMU electrolyte in comparison to previously reported Zn–S cells (based on sulfur weight); f) Nyquist plots with the fitted curves at 20 °C (inset showing the equivalent circuit) and g) linear fits to calculate activation energies; h) cycling performance at 1 A g^−1^.

The electrochemical performance of Zn–S cells with different electrolytes is first evaluated. A 1 m Zn(OAc)_2_+0.1 m ZnI_2_ aqueous solution is applied as the benchmark electrolyte, and TTMU with different percentages is introduced as the additive (labeled as no‐TTMU and x‐TTMU for x% additive, respectively; unit “M” in mol kg_solvent_^−1^). **Figure** **1**a,b compares the charge/discharge and differential capacity curves at the current density of 0.1 A g^−1^. After excluding the capacity contribution from iodide in electrolyte above 1.25 V, the sulfur cathode delivers a discharge capacity of 1138 mAh g^−1^ in the benchmark electrolyte, with a large overpotential of 0.65 V. With the introduction of only 2% TTMU, the sulfur cathode presents the improved capacity of 1219 mAh g^−1^ with 0.45 V overpotential. The battery performance further enhances with the increase of TTMU percentage to 10%, whereas 15% TTMU leads to capacity decay. It demomnstrates 10% as the optimal TTMU percentage, which reaches a high discharge capacity of 1620 mAh g^−1^ accompanied by a low overpotential of 0.37 V. A closer examination reveals that TTMU primarily elevates the discharge plateau. The similar charge plateaus observed in different electrolytes are attributed to the catalyst effects of iodide. Nevertheless, the reversibility of the iodide reaction above 1.25 V is improved upon TTMU addition.

The superiority of the 10‐TTMU electrolyte for Zn–S cells is further examined. Figure 1c,d compares the rate performance with and without the TTMU additive. The cathode suffers rapid capacity decay in the no‐TTMU electrolyte, demonstrating the extremely poor reaction kinetics. In contrast, upon the addition of 10% TTMU, the sulfur cathode delivers much enhanced capacity of 1620, 1462, 1256, 1240, 1158, 1055, 945 and 913 mAh g^−1^ at 0.1, 0.2, 0.5, 1, 2, 3, 4, and 5 A g^−1^, respectively, together with significantly improved reaction reversibility. It ensures a high energy density of 1296 Wh kg^−1^ at the power density of 80 W kg^−1^ and 256 Wh kg^−1^ at 1400 W kg^−1^ (based on sulfur, Figure 1e). The performance is much better than reported Zn–S cells.^[^ ^19^, ^21^, ^22^, ^24^, ^25^, ^27^, ^28^, ^29^, ^30^ ^]^ Besides, both the capacities and overpotentials of our system are also superior to those of previously studied Zn–S cells (Figure S1, Supporting Information). To understand the enhanced reaction activity with the TTMU additive, electrochemical impedance spectroscopy (EIS) is carried out at different temperatures ranging from 20 to 35 °C (Figure S2, Supporting Information). The Nyquist plots are fitted with a typical equivalent circuit shown in the inset, resulting in reduced transfer resistance (*R*_ct_) in the 10‐TTMU system than in the benchmark electrolyte under all conditions. For instance, the sulfur electrode presents the *R*_ct_ value of 27.9 Ω in the TTMU containing electrolyte at 20 °C, which is much lower than 57.9 Ω in the benchmark electrolyte (Figure 1f). The activation energies (*E*_a_) for the charge transfer processes are further evaluated by the linear fits according to the Arrhenius equation at different temperatures (Figure 1g). It is calculated to be 22.11 and 32.40 kJ mol^−1^ in the electrolytes with and without TTMU additive, respectively, implying the enhanced reaction kinetics by TTMU. The cycling performance is evaluated in the two systems, and the TTMU additive effectively improves the stabilities at different current densities (Figure 1h; Figure S3, Supporting Information). For instance, the cell with the benchmark electrolyte shows facile capacity decay at 1 A g^−1^, remaining only 108 mAh g^−1^ after 150 cycles. In contrast, upon TTMU introduction, the remained capacity increases to 449 mAh g^−1^. The results confirm that the TTMU electrolyte additive dramatically promotes the electrochemical performances of Zn–S cells.

**Figure 2:**
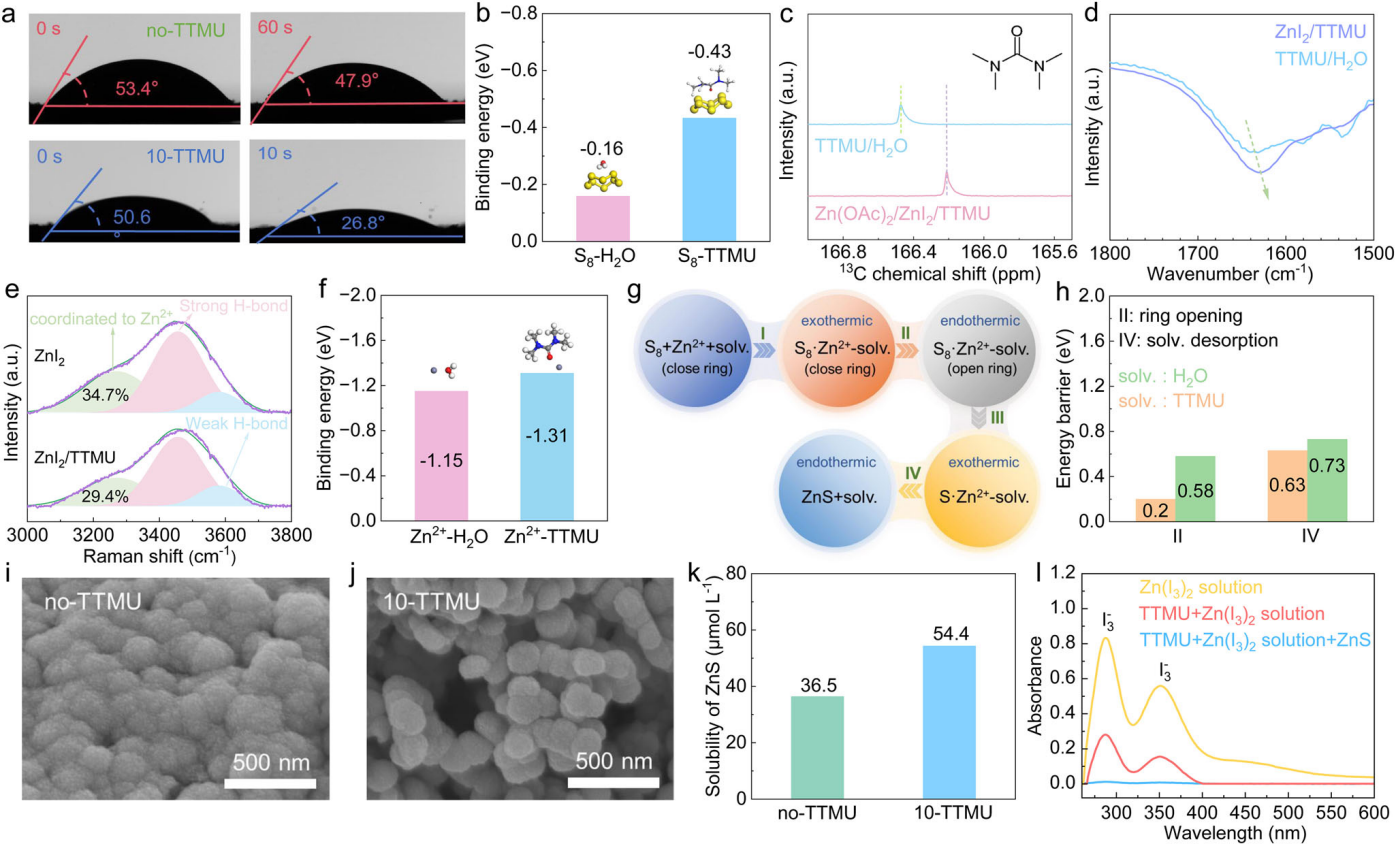
a) Contact angle measurements of sulfur electrode in the no‐TTMU and 10‐TTMU electrolytes. b) Calculated binding energies of S_8_ with H_2_O and TTMU. c) ^13^C NMR and d) FT‐IR of the water‐TTMU mixed solvent, and after adding zinc salts. e) The fittings of O‐H Raman bands in the ZnI_2_ solution without and with TTMU additive. f) Calculated binding energies of Zn^2+^ with H_2_O and TTMU. g) Illustration for the reaction processes of S_8_ with Zn^2+^‐H_2_O or Zn^2+^‐TTMU, and h) the energy barriers of the endothermic steps. SEM images of the discharged cathodes in the i) no‐TTMU and j) 10‐TTMU electrolytes. k) Solubilities of ZnS in the two electrolytes by ICP analysis. l) UV–vis spectra of 100 times diluents of saturated Zn(I_3_)_2_ and TTMU/Zn(I_3_)_2_ solutions, and after the addition of ZnS to the latter.

The functioning mechanism of the TTMU additive is studied. The electrolyte affinities with the sulfur cathode are evaluated by the dynamic contact angles, as illustrated in **Figure** **2**a. The initial contact angle of the TTMU free solution is 53.4°, which decreases to 47.9° after 1 min. Upon the addition of TTMU, the contact angle starts at 50.6° and decreases rapidly to 26.8° within 10 s. It suggests TTMU additive significantly enhances the wettability of the electrolyte on the cathode. The interfacial interaction of sulfur with H_2_O and TTMU is further calculated and compared. As shown in Figure 2b, the S_8_‐TTMU interaction exhibits a stronger binding energy than S_8_‐water, i.e., −0.43 versus −0.16 eV, which explains the smaller contact angles after TTMU introduction. The enhanced wettability helps to reduce the interfacial resistance and facilitate reaction kinetics. Meanwhile, the preferentially adsorbed TTMU can also interact with Zn^2+^ at the interface, which further regulates the reaction pathway of the sulfur cathode.

The modified Zn^2+^ chemical environment with the TTMU additive is investigated. Figure 2c and Figure S4 (Supporting Information) shows the ^13^C nuclear magnetic resonance (NMR) of the water‐TTMU mixed solvent (blue) and after adding zinc salts (pink). The C═O group of TTMU shows up at 166.48 ppm in the solvent, which shifts to 166.21 ppm upon the introduction of zinc salts. It suggests that TTMU is coordinated with the added Zn^2+^. This is further confirmed by Fourier transform infrared (FT‐IR) spectroscopy. Considering the possible signals from the acetate group in Zn(OAc)_2_, 1 m ZnI_2_ with 10% TTMU electrolyte is applied instead. As shown in Figure 2d, the carbonyl peak experiences a redshift from 1636 to 1628 cm^−1^ upon salt addition, demonstrating its coordination with Zn^2+^. The electrolyte structures are further studied by the Raman spectra of 1 m ZnI_2_ in water and after 10% TTMU addition (Figure 2e). The broad band above 3000 cm^−1^ is attributed to the O‐H vibration of water. It is fitted with the different environments of Zn^2+^ coordinated water, strong and weak H‐bonds, with the increase of Raman shift. The fitting reveals the decreased percentage of water coordinated with Zn^2+^ from 34.7% in the 1 m ZnI_2_ solution to 29.4% after 10% TTMU introduction. This is attributed to the coordination of TTMU with Zn^2+^ in the electrolyte, in agreement with ^13^C NMR and FT‐IR results. Molecular dynamics (MD) simulations are further performed to study the solvation structure of Zn^2+^ in the 10‐TTMU electrolyte (Figure S5,Supporting Information). As shown in the radial distribution functions (RDFs), two Zn‐O(H_2_O) peaks at 1.99 and 4.31 Å are detected, corresponding to the first and second solvation shells of H_2_O around Zn^2+^. Meanwhile, the Zn‐O(TTMU) peak at 2.0 Å is monitored, which suggests the entrance of TTMU into Zn^2+^ solvation shells. The regulated solvation structures by TTMU additive result in enhanced electrolyte conductivities at different conditions (Figure S6, Supporting Information). More importantly, it would modulate the reaction properties of the sulfur electrode.

The interaction behaviors and the effects on the sulfur reaction process are analyzed by density functional theory (DFT) calculations. Figure 2f shows the binding energies of −1.15 and −1.31 eV for Zn^2+^‐H_2_O and Zn^2+^‐TTMU, respectively, confirming the preferential coordination of TTMU with Zn^2+^ over water. This regulated Zn^2+^‐solvent interactions affect the subsequent reaction of the sulfur cathode. As shown in Figure 2g,h and Table S1 (Supporting Information), the reaction pathway include the absorption of Zn^2+^‐H_2_O or Zn^2+^‐TTMU with S_8_, the ring opening, the reduction to S^2−^, and the desorption of water or TTMU processes. Specifically, the ring opening and desorption steps are endothermic. Comparing the two systems, the Zn^2+^‐TTMU species presents the energies of 0.2 and 0.63 eV during the above two processes, respectively, lower than 0.58 and 0.73 eV for Zn^2+^‐H_2_O. It suggests that the coordination of TTMU with Zn^2+^ helps to facilitate the reaction with the sulfur cathode, therefore ensuring much‐promoted reaction kinetics in the 10‐TTMU electrolyte.

Figure 2i,j and Figure S7 (Supporting Information) shows the scanning electron microscopy (SEM) images and particle size distributions of the cathode after discharging in the no‐TTMU and 10‐TTMU electrolytes. The obtained ZnS particles aggregate in the baseline electrolyte, with an average diameter of ≈1.1 µm. Upon TTMU introduction, in comparison, the ZnS particles become more dispersed and the average diameter reduces to ≈0.8 µm, suggesting the more uniform nucleation of ZnS during the discharge process. To understand the difference, the solubility of ZnS in the two solutions is measured by inductively coupled plasma (ICP, Figure 2k). The concentration increases from 36.5 to 54.4 µm upon the introduction of TTMU. This difference, together with the reduced reaction barrier discussed above, helps with the nucleation of ZnS and promotes its growth into uniform small particles. It provides more reaction sites and ion transport paths in the cathode, demonstrating another reason for the enhanced reaction activity.

In the Zn–S cells, iodide has been introduced as the redox mediator to catalyze the charge process.^[^ ^19^ ^]^ However, the high solubility of iodine species and the associated shuttling effect result in poor reversibility. As shown in Figure 1a, the TTMU additive also helps with the iodide reversibility and thereby the coulombic efficiency of Zn–S cells. The underlying mechanism is studied by UV–vis spectroscopy. A saturated Zn(I_3_)_2_ solution was prepared by mixing ZnI_2_ and I_2_ in a stoichiometric molar ratio to investigate the effect of TTMU on I_3_^−^. Figure 2l shows the spectra of 100 times diluents of saturated Zn(I_3_)_2_ solutions without and with TTMU additive. Interestingly, the absorbance of the main I_3_^−^ peak in the TTMU‐containing solution is only 1/3 of the one in the TTMU free system. The ZnS powder is further added into the Zn(I_3_)_2_+TTMU solution, and the peaks of I_3_^−^ effectively disappear. The above change in the UV‐spectra of I_3_^−^ would be attributed to the formation of a charge‐transfer complex with TTMU,^[^ ^31^, ^32^ ^]^ which suppresses the shuttling in cells and ensures high coulombic efficiency; nevertheless, it still preserves enough power to chemically oxidize ZnS during the charging process, maintaining its effect as the redox mediator.

**Figure 3:**
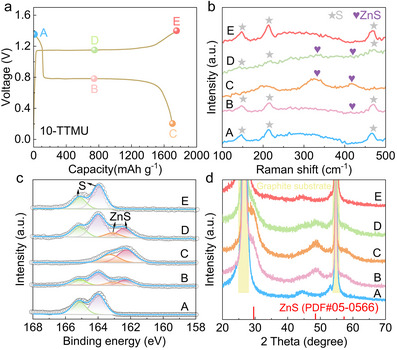
a) The charge/discharge curves of Zn–S batteries at 0.1 A g^−1^ with different spheres indicating the states at which b) Raman, c) XPS, and d) XRD are collected.

The energy storage process of the sulfur cathode in the 10‐TTMU electrolyte is investigated. The Raman, X‐ray photoelectron spectroscopy (XPS), and X‐ray diffraction (XRD) patterns at different states, as labeled in **Figure** **3**a are collected. During discharge, three signals of sulfur at ≈147, 213, and 468 cm^−1^ in the Raman spectrum became weakened and disappeared finally, while the peaks of ZnS at ≈337 and 406 cm^−1^ appear and strenthened gradually, corresponding to the reduction of sulfur to ZnS (Figure 3b).^[^ ^23^ ^]^ In contrast, during the charging process, opposite peak changes are observed, i.e., the diminish of ZnS signals and reappearance of sulfur signals. This demonstrates the highly reversible oxidation of ZnS to S. The reversible process is also verified by XPS analysis. As shown in Figure 3c, the pristine spectrum is fitted by two peaks at 164.1 and 165.1 eV, corresponding to the 2p_3/2_ and 2p_1/2_ splitting of S^0^, respectively. Upon discharge, two more peaks emerge at 162.2 and 163.1 eV, which are the two signals in the fully discharged cathode. They are attributed to the discharged product ZnS.^[^ ^18^ ^]^ The reversible change is presented during charge. Figure 3d shows the evolution of XRD patterns. The pristine cathode presents no extra signals besides the graphite substrate, suggesting the amorphous phase of sulfur. In the discharging process, the peaks at ≈28°, 48°, and 56° grow. They are assigned to ZnS (PDF#05‐0566).^[^ ^21^ ^]^ These signals reversibly disappear during charge, which suggests the electrochemically formed sulfur is also amorphous in our system. Overall, these results confirm the highly reversible redox reaction between S and ZnS in the cell with the TTMU additive.

**Figure 4:**
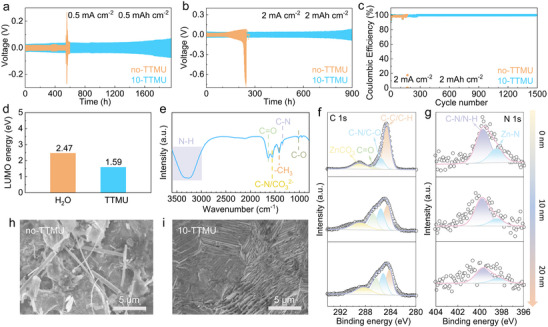
Electrochemical properties of zinc anode in the no‐TTMU and 10‐TTMU electrolytes: cycling performance of Zn||Zn symmetric cells at a) 0.5 mA cm^−2^/0.5 mAh cm^−2^ and b) 2 mA cm^−2^/2 mAh cm^−2^; c) the coulombic efficiency evolutions in Zn||Cu cells at 2 mA cm^−2^/2 mAh cm^−2^. d) The LUMO energy levels of H_2_O and TTMU. e) FT‐IR of Zn electrode after 2 mA cm^−2^ deposition for 1 h in the 10‐TTMU electrolyte. f) The C 1s and g) N 1s XPS of Zn electrode after 100 cycles in the 10‐TTMU electrolyte. SEM images of Zn electrode after 100 cycles in h) no‐TTMU and i) 10‐TTMU electrolytes.

Considering the potential corrosion of halide anions to metal, the stabilities of the Zn anode in ZnI_2_ containing systems are also essential for the electrochemical performance of Zn–S cells. This is evaluated by long‐term plating/stripping in Zn||Zn symmetric cells. As shown in **Figure** **4**a,b, a sudden increase of voltage polarization appears after 550 h in the baseline electrolyte at 0.5 mA cm^−2^ and 0.5 mAh cm^−2^, and the cycle time further decreases to 230 h as the current density and capacity increase to 2 mA cm^−2^ and 2 mAh cm^−2^. Upon the introduction of the TTMU additive, a significantly improved stability is obtained for the Zn electrode. The cycling time extends to over 1900 and 900 h, respectively, in the above two conditions. The reversibility of Zn plating/stripping is further studied in Zn||Cu cells (Figure 4c; Figure S8, Supporting Information). The cell without TTMU failed after 168 cycles, which is attributed to dendrite‐induced short circuit. On the contrary, the TTMU additive enables a stabilized coulombic efficiency of 99.85% for over 1500 cycles. The results demonstrate that TTMU also effectively enhances the reversibility and stability of Zn plating/stripping at the anode.

The effect mechanism of the TTMU additive on Zn stabilities is studied. Figure 4d compares the calculated energy levels of the lowest unoccupied molecular orbitals (LUMOs) of water and TTMU. The latter presents a lower level of 1.59 eV, indicating its reduction capability to generate SEI on the Zn electrode. The surface composition of the Zn electrode after 2 mAh cm^−2^ deposition in the 10‐TTMU electrolyte is characterized by FT‐IR spectroscopy (Figure 4e). The vibration bands from C─O, C─N, ─CH_3_, C─N/CO_3_^2−^, C═O, and N─H functional groups are identified at 1019, 1346, 1414, 1553, 1635, and 3000–3600 cm^−1^, respectively,^[^ ^33^, ^34^ ^]^ resulting from the decomposition of TTMU. Figure 4f,g shows the XPS analysis of the cycled electrode. The C 1s spectrum is fitted with ZnCO_3_, C═O, C─N/C─O, and C─C/C─H,^[^ ^35^ ^]^ and their intensities gradually weaken upon sputtering. In the N 1s spectra, peaks for C─N/N─H and Zn─N are observed,^[^ ^36^ ^]^ together with similar intensity decay after sputtering. The results demonstrate that an organic–inorganic hybrid SEI layer is formed on the surface of Zn. Figure 4h,i presents the SEM images of Zn electrode cycled in the two electrolytes. An uneven surface is obtained after cycling in the baseline electrolyte, whereas the TTMU additive enables the formation of a densely packed surface. The uniform deposition of the latter is attributed to the homogeneous Zn^2+^ flux and anti‐corrosion properties provided by the effective SEI. Overall, these findings confirm that the TTMU electrolyte additive simultaneously enhances the reaction kinetics of the S cathode and stability of the Zn anode, ensuring the excellent electrochemical performance of Zn–S cells.

## 3 Conclusion

In conclusion, we present an interface chemistry and reaction pathway modification strategy by TTMU electrolyte additive to simultaneously facilitate the reaction kinetics of sulfur cathode and enhance the stability of Zn anode in aqueous Zn–S cells. Combined experimental and theoretical analysis demonstrate that TTMU preferentially adsorbs on the sulfur surface and interacts with Zn^2+^, which alters their following reaction process. It reduces the energy barriers and regulates the nucleation/growth behavior, therefore enhancing the reaction kinetics of the sulfur cathode. Besides, the TTMU additive also suppresses the shuttling of iodine redox mediator to ensure high reaction reversibility. Thanks to these effects, the sulfur cathode delivers a high capacity of 1620 mAh g^−1^ at 0.1 A g^−1^ with a low overpotential of 0.37 V in the electrolyte containing 10% TTMU additive, superior to 1138 mAh g^−1^/0.65 V obtained in the benchmark electrolyte. It also maintains a good capacity of 913 mAh g^−1^ as the current density increases to 5 A g^−1^ with the additive, which again far exceeds 48 mAh g^−1^ in the benchmark electrolyte. Moreover, an organic–inorganic hybrid SEI layer is induced on the zinc surface by the TTMU additive, which enhances the stability and reversibility of Zn plating/stripping. Therefore, the Zn electrode exhibits stable cycling for over 1900 h in symmetric cells and achieves a high coulombic efficiency of 99.85% for more than 1500 cycles. This study provides key insights into the reaction path regulation of Zn–S cells to promote the redox kinetic and cycling stability. It would inspire further strategies to control the detailed energy storage mechanisms on both electrodes and promote the electrochemical performance of aqueous Zn–S batteries, as well as other conversion‐type electrodes.

## 4 Experimental Section

### Materials

Graphite foil was purchased from SGL group (Germany). Sulfur and sodium alginate were purchased from Sinopharm Chemical Reagent Co. Ltd. Ketjen black (KB) was purchased from Lion Specialty Chemicals (Japan). Iodine (I_2_), zinc acetate (Zn(OAc)_2_), zinc iodide (ZnI_2_) and tetramethylurea (TTMU) were obtained from Aladdin Chemical Reagent Co. Ltd. Glass fiber separators were purchased from Merck Millipore.

### Material Characterizations

Thermogravimetric analysis (TGA) was carried out on a Ruigaku TG/DTA8122 (Japan) at temperatures ranging from 30 to 500 °C with a heating rate of 10 °C min^−1^ under an air atmosphere. X‐ray photoelectron spectroscopy (XPS) was performed on a KRATOS, Axis Ultra DLD (UK) using Al‐Kα radiation as the excitation source. The data was analyzed using CasaXPS software, and the C 1s peak was calibrated to 284.5 eV. The morphologies were investigated by HITACHI SU 8010 scanning electron microscope (SEM). X‐ray diffraction (XRD) was carried out on a PANalytical Empyrean diffractometer with Cu Kα radiation. Contact angles were measured on JY‐82. ^13^C nuclear magnetic resonance (NMR) were obtained on a Bruker 600 MHz (Germany). Fourier transform infrared (FT‐IR) spectroscopy was carried out on VERTEX70 (Bruker). UV–vis spectra were carried out by a Lambda XLS+ UV–vis spectrometer. Raman was obtained on BWS465‐532S, B&W Tek Inc., USA, with an excitation wavelength of 532 nm. The ZnS solubilities in electrolytes were measured by inductively coupled plasma optical emission spectroscopy (ICP‐OES) on a PerkinElmer 8300 instrument. Particle sizes were measured by a Nanoparticle size analyzer (Nano‐S90).

### Electrochemical Measurements

The sulfur active material was first mixed with KB at a mass ratio of 5:4 at 155 °C for 10 h. It was then mixed with sodium alginate in water at a mass ratio of 9:1 (5:4:1 ratio of S:carbon: binder). The slurry was cast onto a graphite foil substrate and dried at room temperature. The mass loading of sulfur was 2–2.5 mg cm^−2^. The diameter of the sulfur cathode and Zn anode was ≈12 mm, and the amount of electrolyte used in the cell was 80 µL. Zn–S cells were assembled with two‐electrode Swagelok cells. Electrochemical impedance spectroscopy (EIS) was carried out in the frequency range from 100 mHz to 100 kHz. Electrochemical properties of Zn||Zn symmetric and Zn||Cu asymmetric cells were tested in CR2032 coin cells. All electrochemical measurements were carried out using LAND (CT2001A) or Bio‐logic VMP3 multichannel electrochemical systems.

### Theoretical Calculations

The density functional theory (DFT) calculations of binding energies and molecular orbitals were performed using the Gaussian16 software. Structure optimizations were performed at the B3LYP/6–31G (d,p) level. The binding energies (*E*) between different species were calculated by the following equation:

(1) E=E(A+B)−E(A)−E(B)

*E_(A+B)_*, *E_(A),_* and *E_(B)_* were the energies of the interacted structures and either species, respectively.

For molecular dynamic (MD) simulations, the box was constructed with the size of 26× 26 ×26 nm^3^, and the molar ratio of Zn(OAc)_2_, ZnI_2_, H_2_O, and TTMU was 100: 10: 5050: 70. NVT was carried out at a constant temperature of 298 K to ensure the equilibrium state for 50 ps, and NPT running for 50 ps with ordinary pressure was applied to study the properties of the structure.

## Conflict of Interest

The authors declare no conflict of interest.

## Supporting information



Supporting Information

## Data Availability

The data that support the findings of this study are available from the corresponding author upon reasonable request.
