# Supplementary material for: Interface Chemistry and Reaction Pathway Regulation for Boosted Redox Kinetics in Aqueous Zn–S Batteries
Source: Adv Sci (Weinh). 2025 Nov 5;13(4):e13155. doi: 10.1002/advs.202513155 (PMC12822381; doi:10.1002/advs.202513155)
Supplement: Supplementary file 1 — Supporting Information [file ADVS-13-e13155-s001.pdf]

Supporting Information

Interface Chemistry and Reaction Pathway Regulation for Boosted Redox Kinetics in Aqueous Zn-S Batteries

Supporting Figures and Table

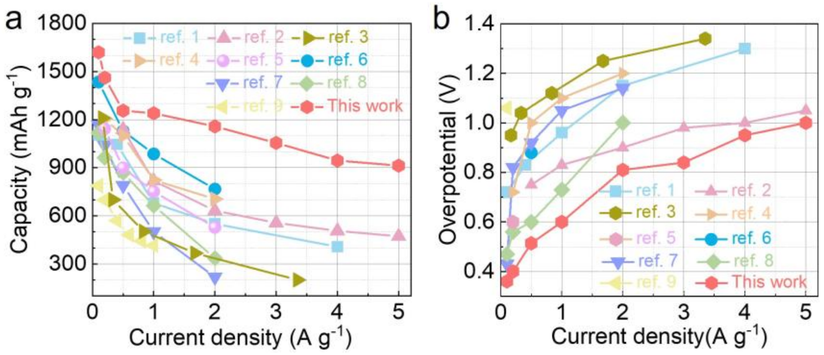

Figure S1. Comparisons of capacity (a) and overpotential (b) in the 10-TTMU electrolyte with previously reported Zn-S cells.

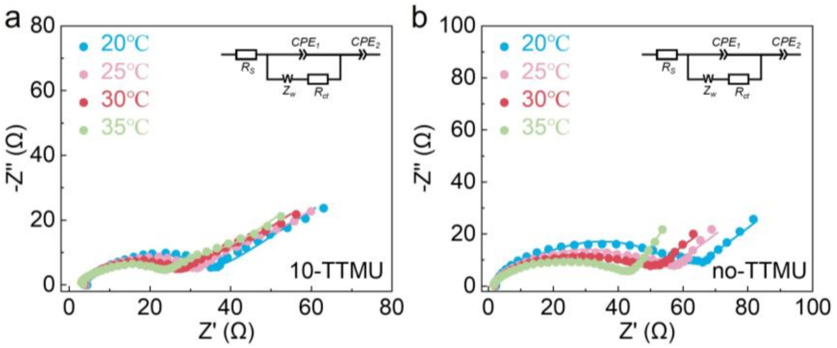

Figure S2. Nyquist plots and fitted curves of sulfur electrode at different temperatures with a) the 10-TTMU and b) benchmark electrolytes (inset showing the equivalent circuit).

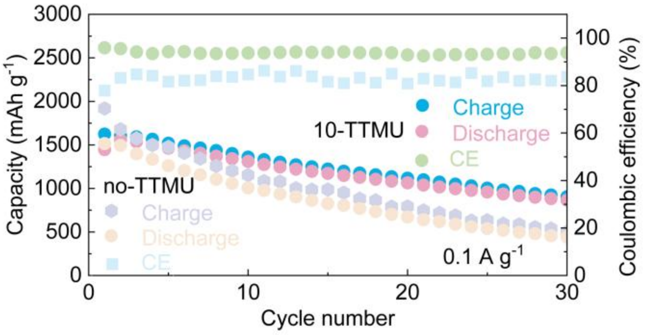

Figure S3. The cycling performance in no-TTMU and 10-TTMU electrolytes at 0.1 A g^{-1}.

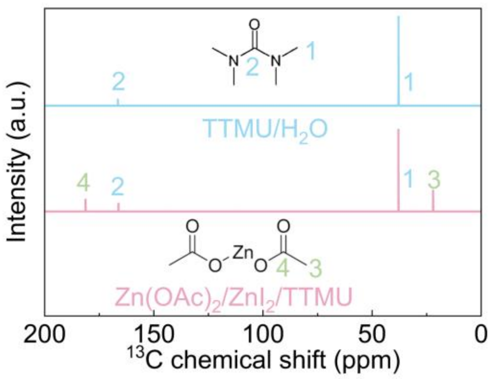

Figure S4. ^{13}C NMR of the water-TTMU mixed solvent and after adding zinc salts.

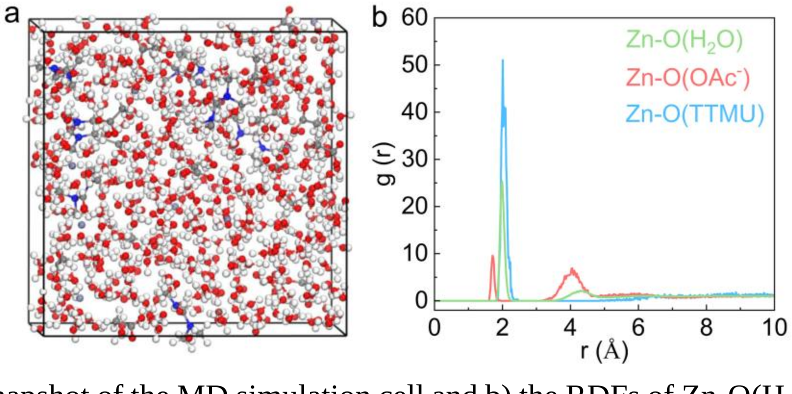

Figure S5. a) Snapshot of the MD simulation cell and b) the RDFs of Zn-O(H_2O), Zn-O(OAc^-) and Zn-O(TTMU).

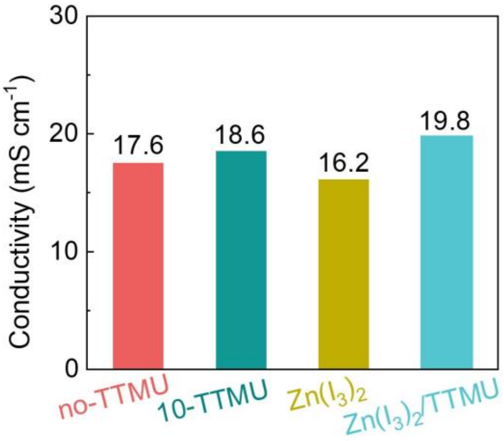

Figure S6. The conductivities of no-TTMU, 10-TTMU, $Zn(I_3)_2$ and $Zn(I_3)_2$/TTMU solutions.

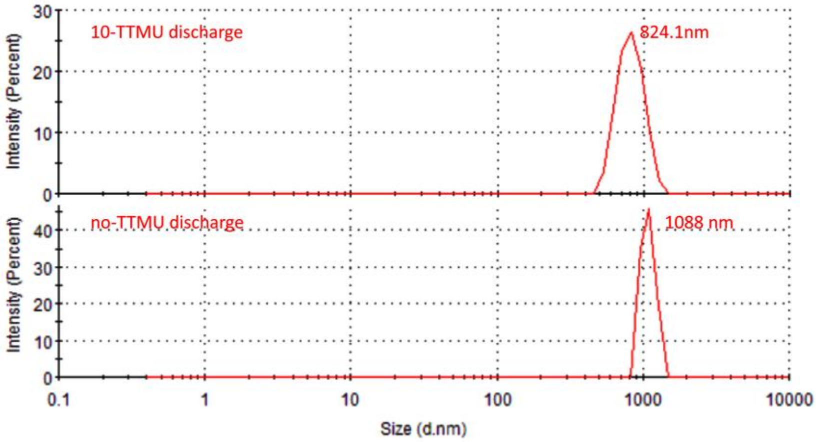

Figure S7. Particle size analysis of the discharged cathode in the two electrolytes.

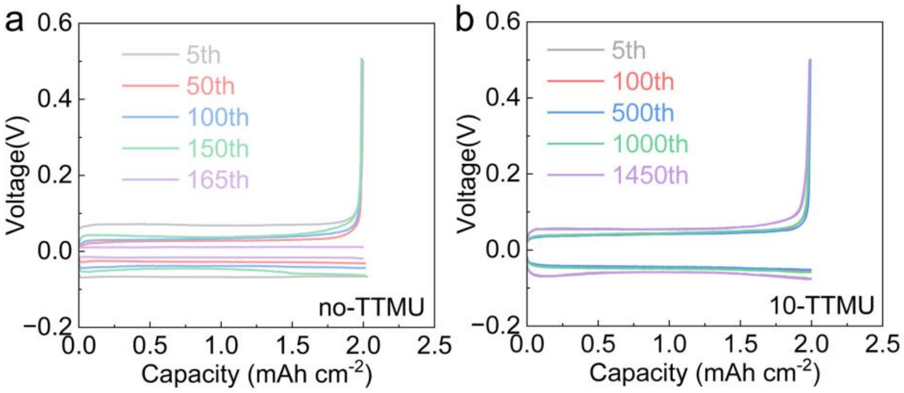

Figure S8. The voltage curves in Zn||Cu cells at 2 mA cm^{-2}/2 mAh cm^{-2} with the two electrolytes.

Table S1 The free energies at each step during the reaction of S_8 with Zn^{2+}-H_2O or Zn^{2+}-TTMU.

Configuration \ Free energy (eV)	Zn^{2+}-H_2O	Zn^{2+}-TTMU
S_8+Zn^{2+}-H_2O/TTMU	0	0
S_8·Zn^{2+}-H_2O/TTMU (close ring)	-4.97	-3.50
S_8·Zn^{2+}-H_2O/TTMU (open ring)	-4.39	-3.30
S·Zn^{2+}-H_2O/TTMU	-22.17	-17.79
ZnS+H_2O/TTMU	-21.44	-17.16

References:

[1] W. Li, K. Wang, K. Jiang, *Adv. Sci.* **2020**, *7*, 2000761.

[2] M. Yang, Z. Yan, J. Xiao, W. Xin, L. Zhang, H. Peng, Y. Geng, J. Li, Y. Wang, L. Liu, Z. Zhu, *Angew. Chem. Int. Ed.* **2022**, *61*, e202212666.

[3] S. Mehta, S. Kaur, M. Singh, M. Kumar, K. Kumar, S. K. Meena, T. C. Nagaiah, *Adv. Energy Mater.* **2024**, *14*, 2401515.

[4] H. Zhang, Z. Shang, G. Luo. S, Jiao. R. Cao, Q. Chen, K. Lu, *ACS Nano* **2021**, *16*, 7344.

[5] W. Zhang, M. Wang, J. Ma, H. Zhang, L. Fu, B. Song, S. Lu, K. Lu, *Adv. Funct. Mater.* **2023**, *33*, 2210899.

[6] Y. Guo, R. Chua, Y. Chen, Y. Cai, E. J. Js. Tang, J. J. N. Lim, T. H. Tran, V. Verma, M. W. Wong, M. Srinivasan, *Small* **2023**, *19*, 2207133.

[7] D. Patel, A. Dharmesh, Y. Sharma, P. Rani, A. K. Sharma, *Chem. Eng. J.* **2024**, *479*, 147722.

[8] T. Zhou, H. Wan, M. Liu, Q. Wu, Z. Fan, Y. Zhu, *Mater. Today Energy* **2022**, *27*, 101025.

[9] Z. Xu, Y. Zhang, W. Gou, M. Liu, Y. Sun, X. Han, W. Sun, C. Li, *Chem. Commun.* **2022**, *58*, 8145-8148.
